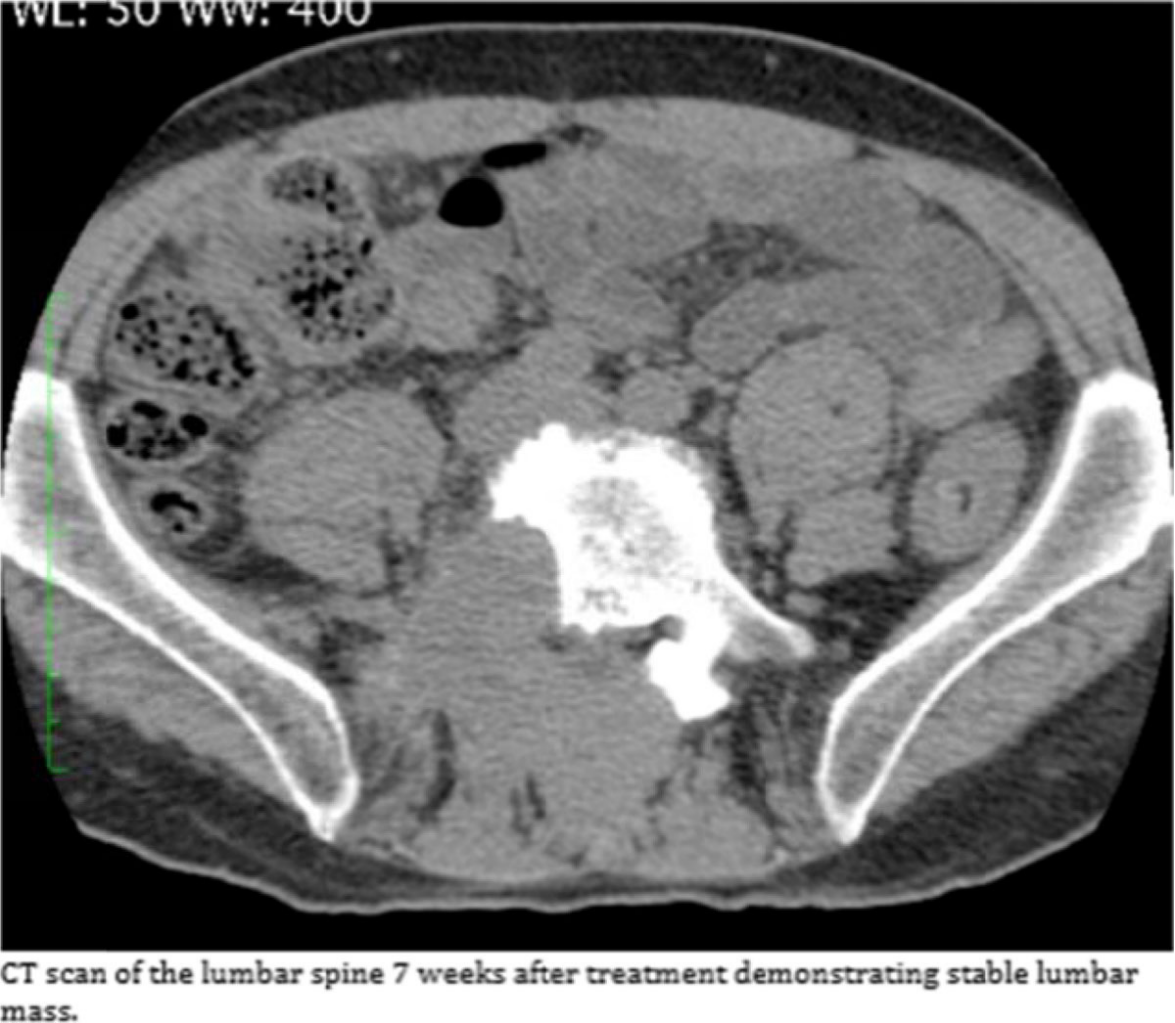# Image guided cryoablation of cancer with intra-tumoral injection of anti-CTLA-4 and PD-1 immune check-point inhibitors

**DOI:** 10.1186/2051-1426-3-S2-P142

**Published:** 2015-11-04

**Authors:** Mark A Rosenberg, Jason Williams

**Affiliations:** 1 Cancer Immune Biologics, Boca Raton, FL, USA; 2 Cancer Immune Biologics, Dunwoody, GA, USA

## Background

Image guided cryoablation of cancer destroys tumor tissue and can activate tumor-specific T cells by increasing the presentation of tumor antigens and causing the release of “Danger signals” to stimulate the immune system. However, the development of a systemic anti-cancer immune response may be restrained by immune check-point inhibitors. In recent years, the U.S. Food and Drug Administration–approved antibody drug ipilumimab, nivolumab and pembrolizumab as inhibitors of CTLA-4 and PD-1. By blocking these immune check-point receptors, these antibodies can promote tumor rejection, but their full application has yet to be fully determined.

## Methods

In our human case studies, we offer a proof-of-concept in multiple tumor types that intra-tumoral CTLA-4 and PD-1 blockade combined with cryoablation of a primary tumor can cause regression of secondary tumors at a distant site.

## Results

From past clinical experience, we know that secondary tumors are unlikely to be affected by cryoablation alone, the combination treatment was sufficient to cause complete cancer regression and tumor rejection. Cryoablation is currently used to treat a targeted tumor, our results suggest that combination therapy with CTLA-4 and PD-1 blockade, by direct injection into the ablated tumor, will enhance anti-tumor immunity and rejection of tumor metastases.

**Figure 1:**
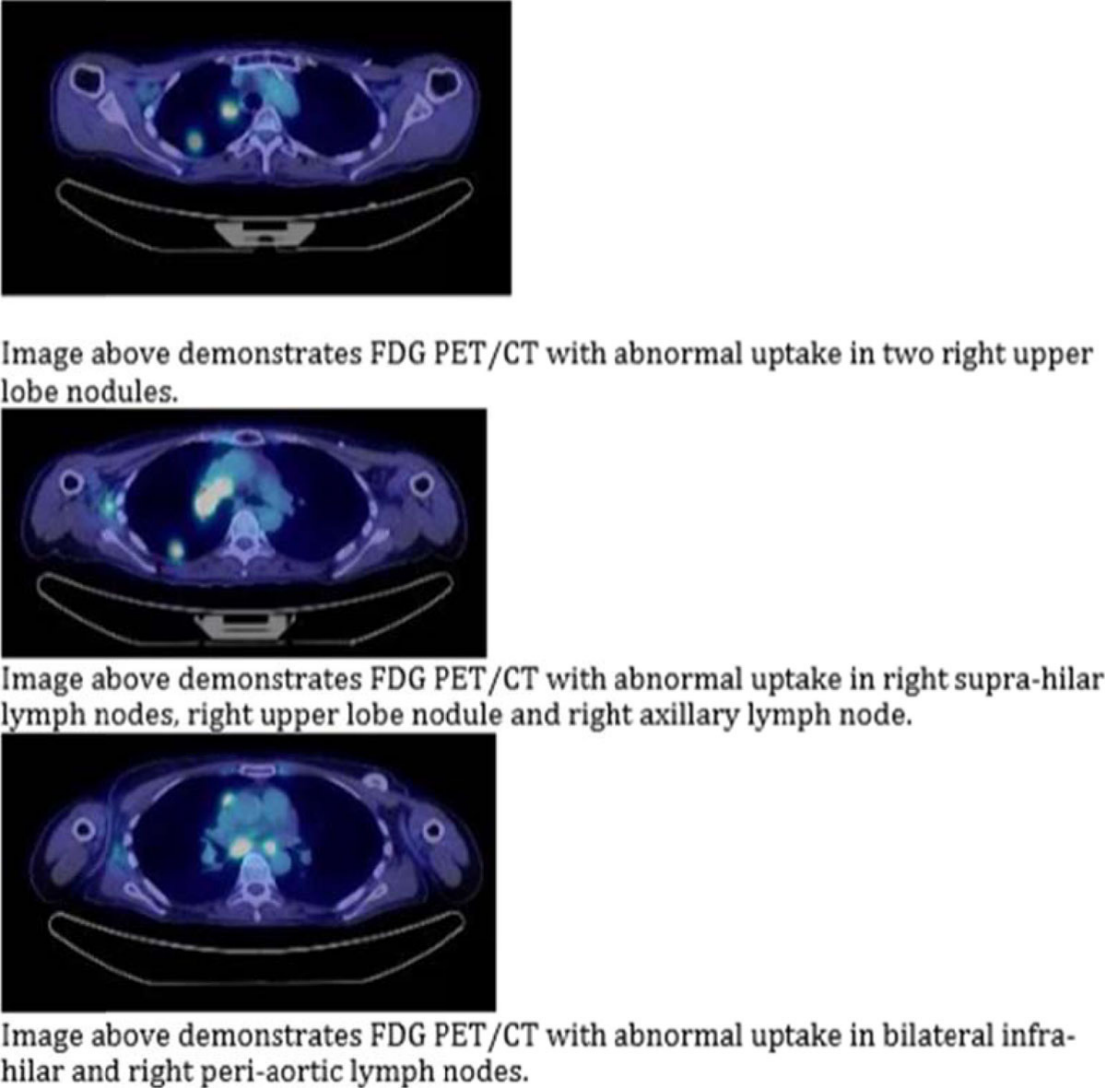


**Figure 2:**
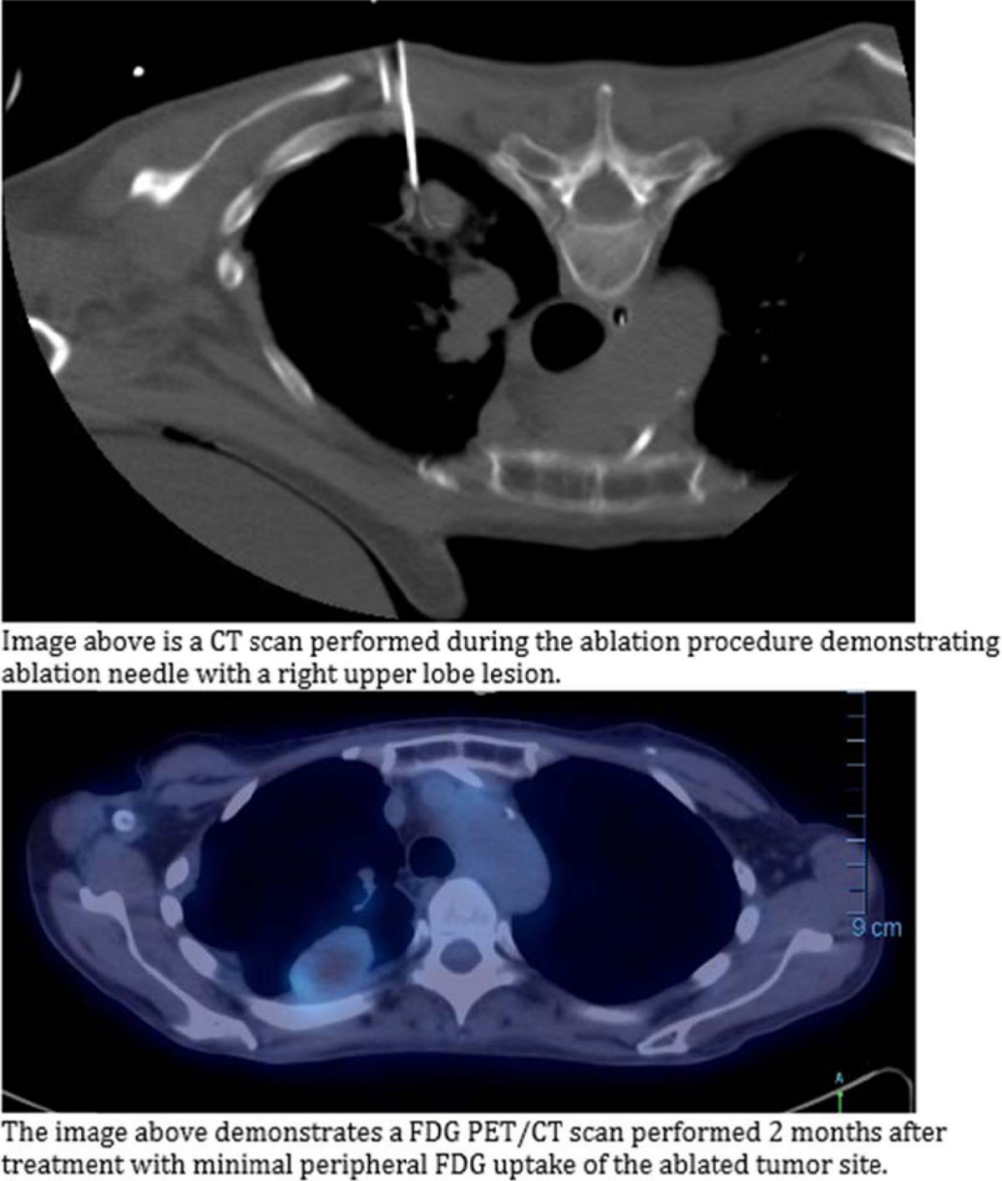


**Figure 3:**
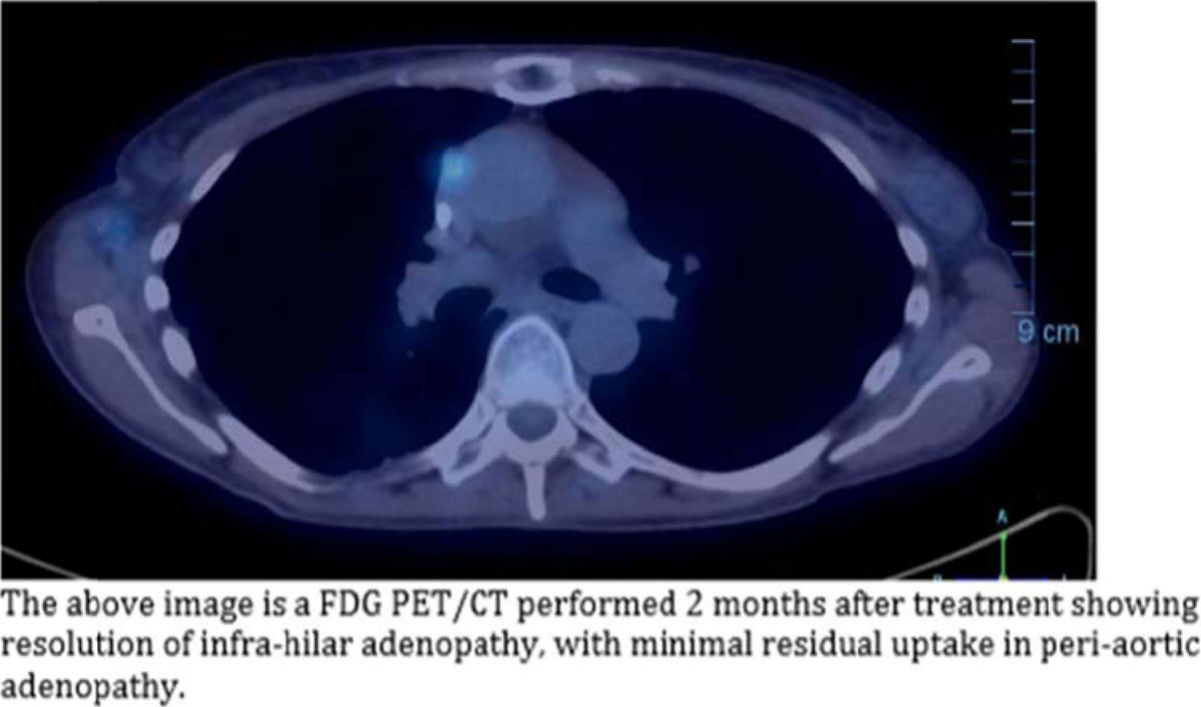


**Figure 4:**
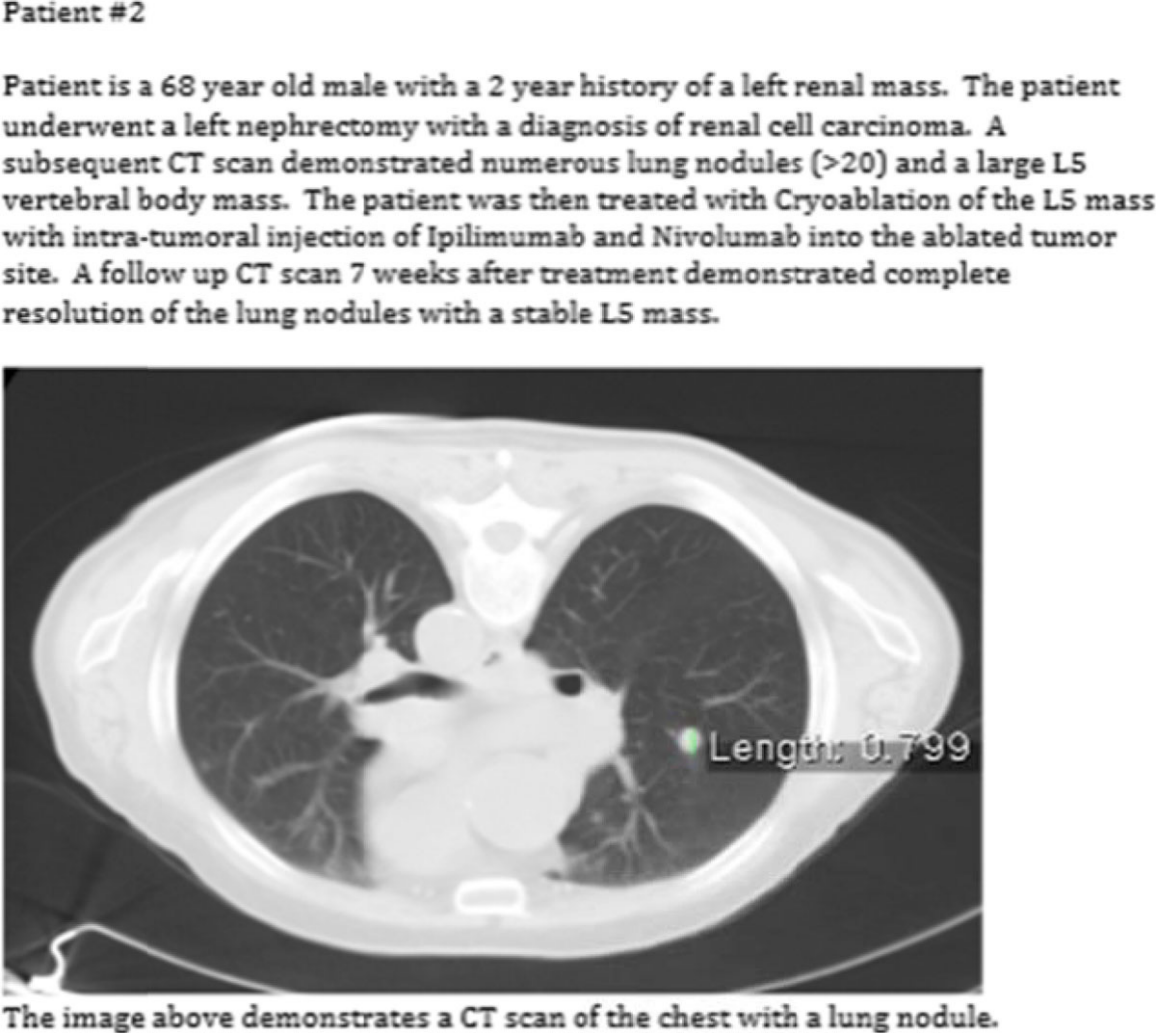


**Figure 5:**
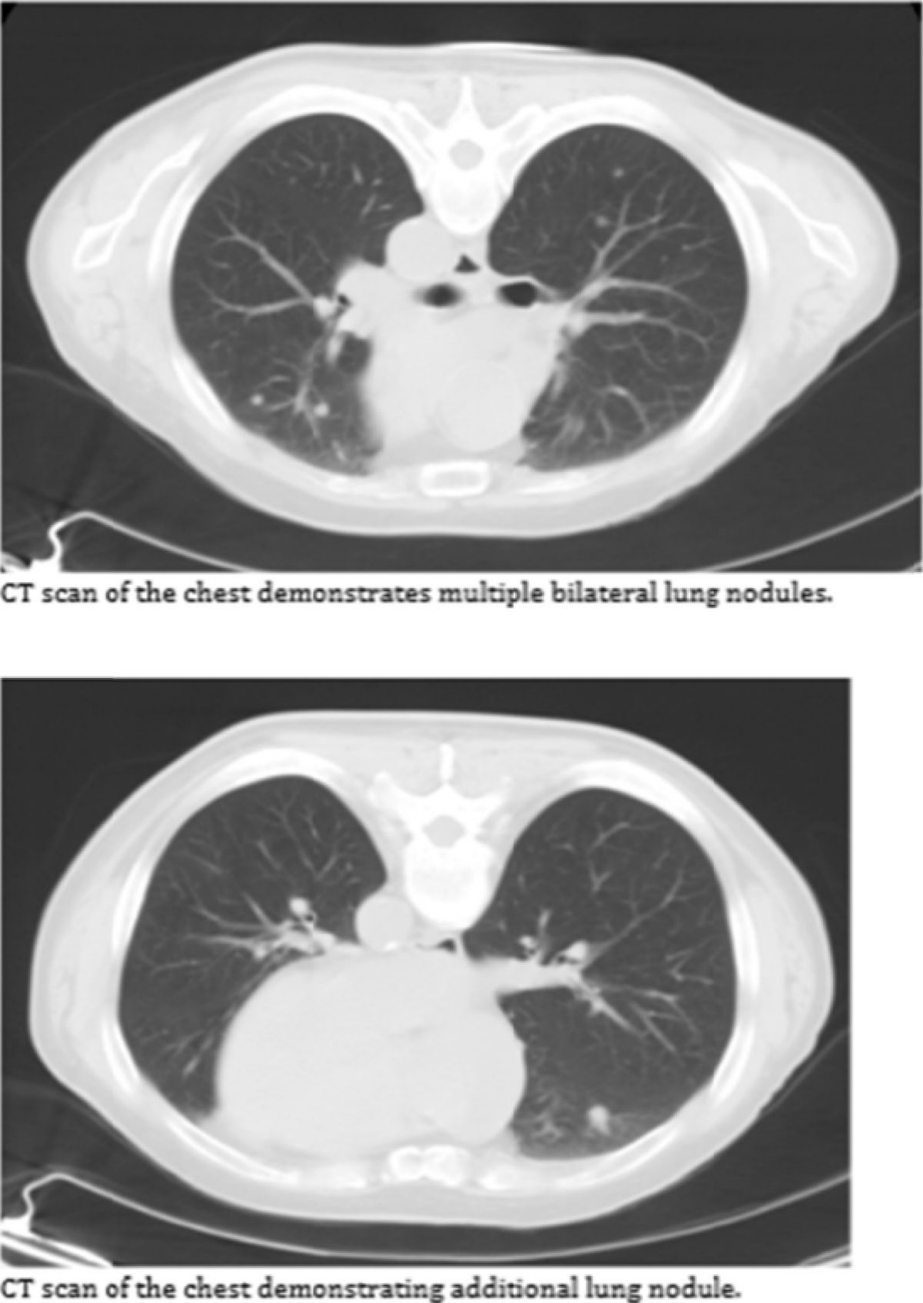


**Figure 6:**
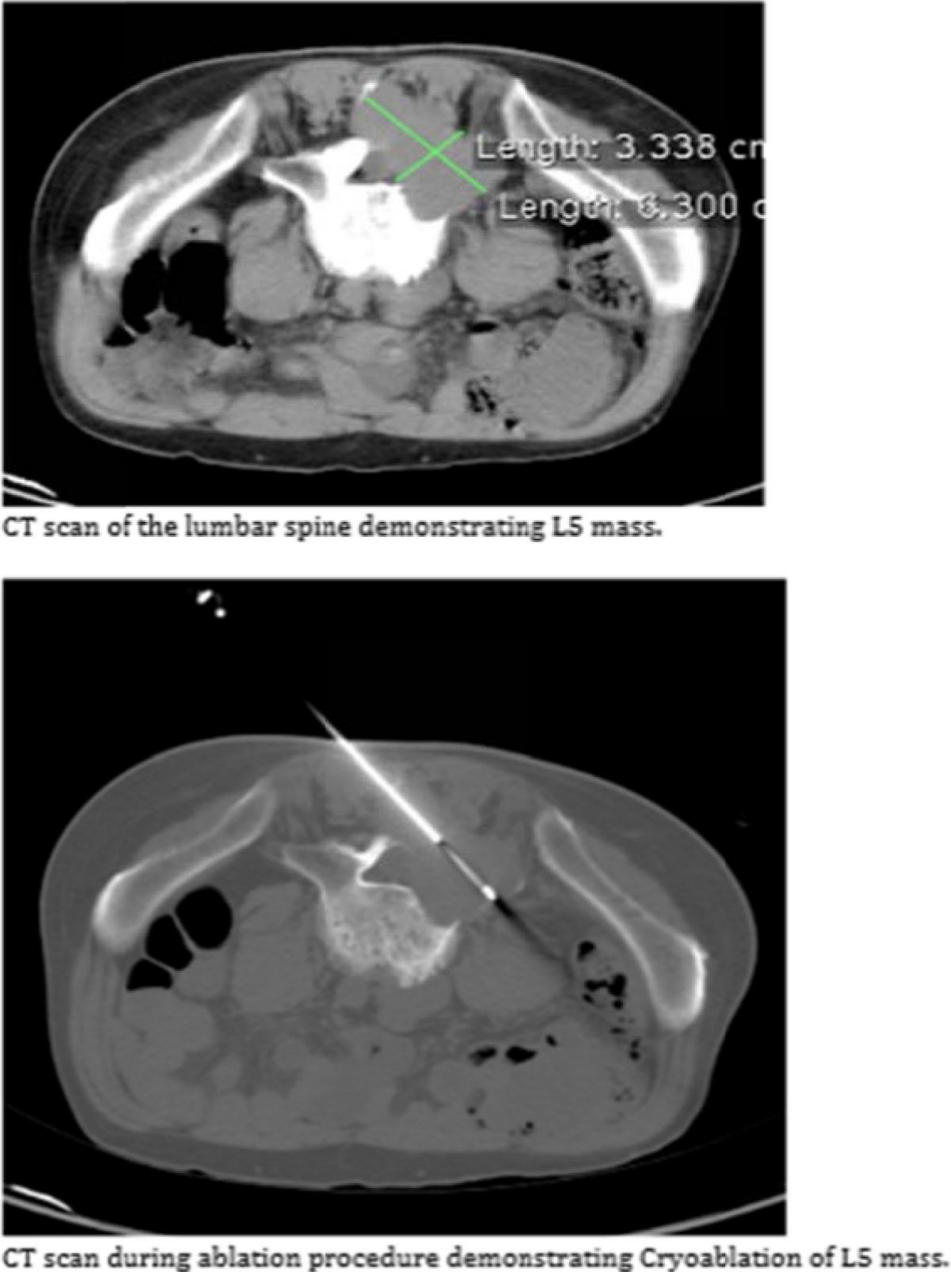


**Figure 7:**
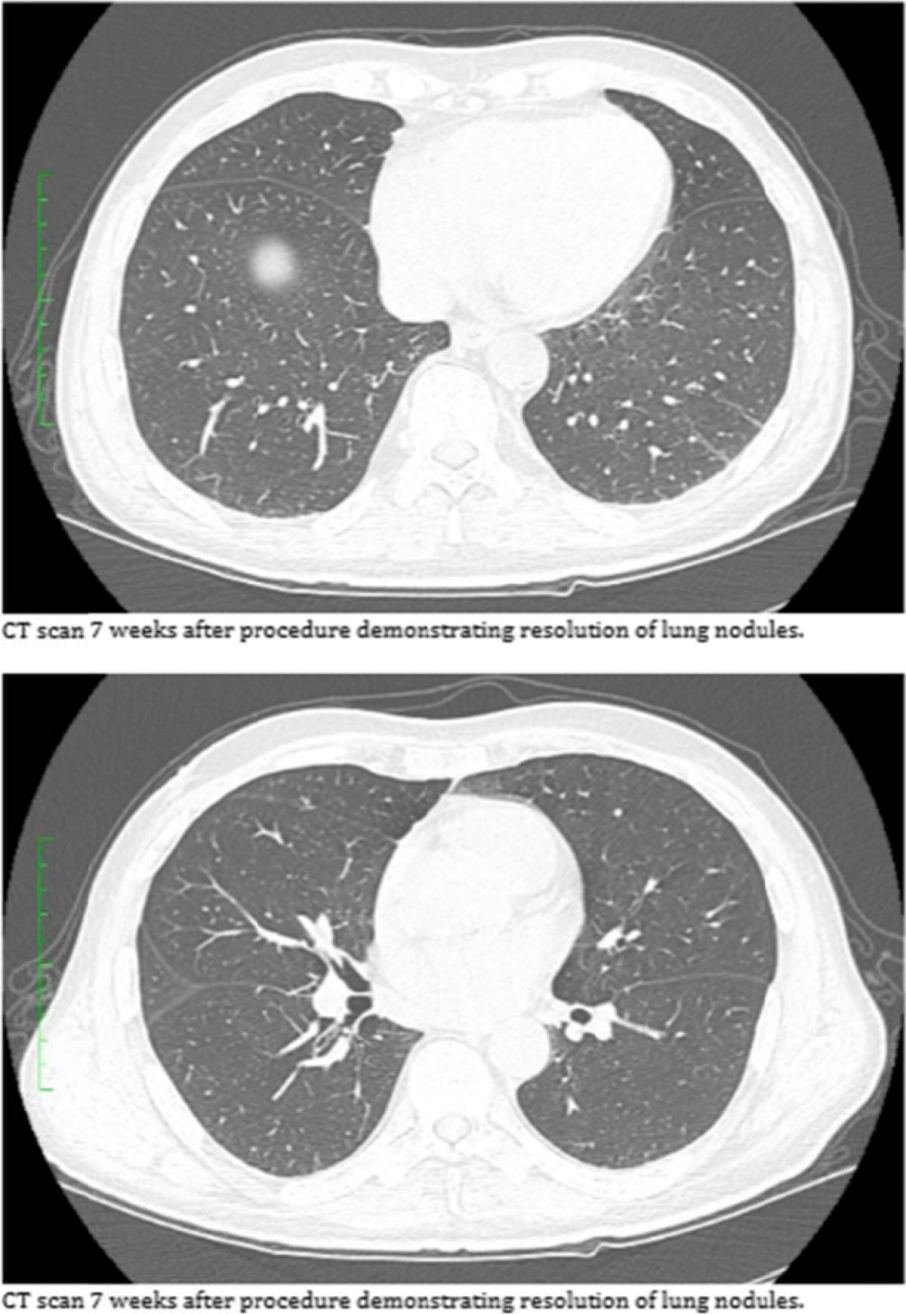


**Figure 8:**
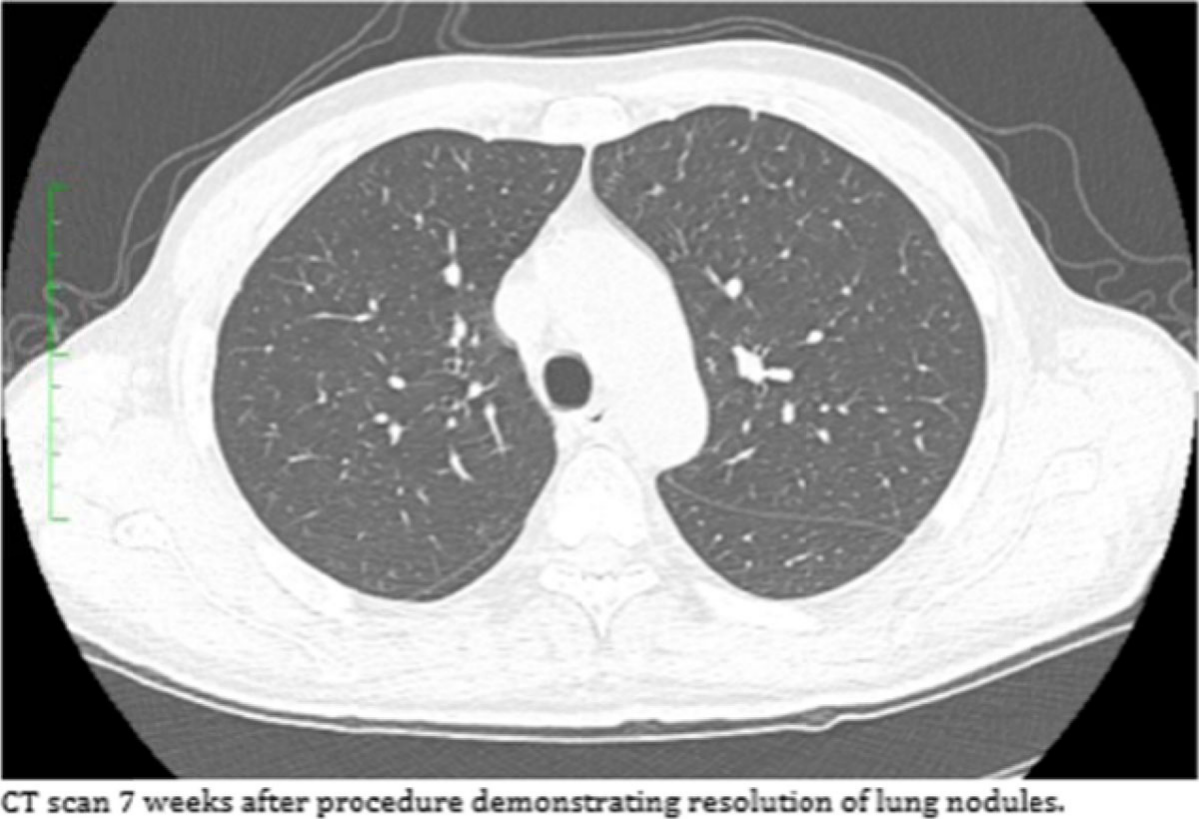


**Figure 9:**